# Lactylation associated biomarkers and immune infiltration in aortic dissection

**DOI:** 10.1038/s41598-025-08613-y

**Published:** 2025-07-01

**Authors:** Jianfeng Ye, Yuntao Fu, Yuanjia Ke, Huanting Liu, Sini Fang, Yumeng Lei, Ying Mao, Liqiu Yan, Youcheng Wang

**Affiliations:** 1 Department of Cardiology, The Affiliated Dongguan Songshan Lake Central Hospital, Guangdong Medical University, Dongguan, 523326 Guangdong China; 2 Dongguan Key Laboratory of Cardiovascular Aging and Myocardial Regeneration, Dongguan Cardiovascular Research Institute, Dongguan, China; 3 Department of Cardiology, Cardiovascular Research Institute, Renmin Hospital of Wuhan University, Wuhan University, Wuhan, China

**Keywords:** Aortic dissection, Protein lactylation, PGK1 and HMGA1, Immune infiltration, Machine learning, Computational biology and bioinformatics, Cardiovascular biology

## Abstract

Protein lactylation, a novel post-translational modification (PTM), has emerged as a critical factor in disease processes related to glycolysis and immune responses. However, its role in aortic dissection (AD) has yet to be thoroughly investigated. This study aimed to investigate the involvement of protein lactylation in AD and identify key lactylation-related genes as potential diagnostic biomarkers. Transcriptomic data from public databases were analyzed to identify differentially expressed lactylation-related genes in AD. Functional enrichment analyses were performed, and Weighted Gene Co-expression Network Analysis (WGCNA) was utilized to identify gene modules associated with AD. Machine learning methods, including LASSO and Random Forest, were employed to identify key diagnostic genes. Experimental validation was performed using human aortic tissues and an AD model. Bioinformatics analysis identified 11 lactylation-related differentially expressed genes (LR-DEGs) in AD. WGCNA and machine learning revealed two optimal feature genes, PGK1 and HMGA1, which were validated in an independent dataset and demonstrated high diagnostic accuracy (AUC: PGK1 = 1, HMGA1 = 0.94). Immune infiltration analysis indicated significant correlations between these genes and specific immune cell types, suggesting a role in immune regulation. Experimental validation in human and murine AD tissues confirmed the upregulation of PGK1 and HMGA1. This study underscores the importance of lactylation in the pathogenesis of AD and identifies PGK1 and HMGA1 as key biomarkers related to lactylation. These findings enhance our understanding of the metabolic and immune mechanisms involved in AD, thereby presenting new molecular targets for diagnosis and therapeutic intervention.

**Supplementary Information:**

The online version contains supplementary material available at 10.1038/s41598-025-08613-y.

## Introduction

Aortic dissection (AD) represents a *life-threatening* cardiovascular emergency with an incidence of approximately 2–3 cases per 100,000 individuals annually^1^. The pathogenesis of AD is multifaceted, involving a complex interplay of genetic, environmental, and hemodynamic factors^2^. *According to the Stanford classification*,* AD is categorized into Stanford type A (involving the ascending aorta) and type B (limited to the descending aorta)*^3^. The clinical manifestations of AD are varied, with common symptoms including severe chest pain, back pain, abdominal pain, and, in some instances, syncope, shock, or other serious complications^4^. The diagnosis of AD *predominantly* relies on imaging modalities, particularly Computed Tomography Angiography (CTA)^5^. In terms of treatment, type A AD typically necessitates surgical intervention, whereas type B AD is primarily managed through medical treatment; however, some patients may require *endovascular interventions depending on complications*. The prognosis for AD is notably poor, with a mortality rate of approximately 1% per hour. Within 24 h of onset, the mortality rate can escalate to 25%, and within one week, it may rise to 50%^6^. Therefore, conducting in-depth research on the pathogenesis of AD and identifying effective diagnostic biomarkers and therapeutic targets are of significant importance for improving patient outcomes.

In recent years, the advancement of omics technologies has led to a heightened focus on the role of post-translational modifications (PTMs) in cardiovascular diseases^7,8^. PTMs are defined as the addition or removal of specific chemical groups on proteins following their synthesis, facilitated by enzyme-catalyzed reactions, which subsequently alters the protein’s structure and function. These modifications are diverse, encompassing phosphorylation, ubiquitination, acetylation, and others, and they play critical roles in cellular signal transduction, protein stability, and the regulation of the cell cycle^9,10^. A growing body of research indicates that the abnormal regulation of PTMs is closely associated with the onset and progression of cardiovascular diseases. Lactylation, a recently discovered PTM, refers to the addition of lactate molecules to the lysine residues of proteins, resulting in the formation of lactylated lysine^11^. Lactylation was first identified in tumor cells, and studies have shown its significant role in tumorigenesis, metastasis, and immune evasion^12^. In recent years, research on lactylation in cardiovascular diseases has increased significantly, although the exact mechanisms remain unclear^13^. Lactylation can alter the conformation, stability, and activity of proteins, thereby affecting their functions^14^. Studies have shown that lactylation regulates the functions of various proteins, including histones, transcription factors, and enzymes^15,16^. *Recent studies have highlighted the role of lactylation in cardiovascular diseases. For instance*,* lactylation has been implicated in the regulation of inflammatory responses and vascular remodeling*,* processes that are critical in the pathogenesis of aortic dissection. Specifically*,* lactylation can modulate the function of immune cells*,* such as macrophages*,* influencing their polarization and activity*,* thereby affecting inflammation and immune surveillance*^17,18^. In aortic dissection, lactylation may influence the onset and progression of the disease by modulating the functions of key proteins.

The immune response serves as a crucial defense mechanism of the body against injury and infection. In the context of cardiovascular diseases, *this response plays a dual role—on one hand*,* it contributes to tissue repair and pathogen clearance; on the other*,* excessive or dysregulated immune activation can exacerbate vascular damage and promote disease progression*^19^. *Increasing evidence has shown that the activation of immune cells—particularly macrophages and T cells—as well as the secretion of pro-inflammatory cytokines (e.g.*,* IL-6*,* TNF-α)*,* are closely linked to the initiation and advancement of cardiovascular pathologies*,* including atherosclerosis*,* myocardial infarction*,* and aortic dissection*^20,21^. Lactylation modification plays an important role in regulating the immune response. Studies indicate that lactylation can influence the function of immune cells, including the polarization and activation of macrophages, thereby impacting inflammation and immune surveillance^22,23^. *For instance*,* histone lactylation has been implicated in promoting the expression of genes associated with tissue repair and anti-inflammatory responses in macrophages*,* suggesting a dynamic role in modulating inflammation and immune homeostasis*^24^. Despite these emerging insights, the role of lactylation in AD remains largely unexplored. This study aims to bridge this knowledge gap by investigating the role of lactylation modification in the pathogenesis of AD and its interaction with immune processes through an integrative bioinformatics and experimental approach. First, bioinformatics techniques will be used to identify differentially expressed lactylation-modified proteins associated with AD. Second, the expression of these proteins will be experimentally validated. Finally, the immune regulatory functions of these proteins in AD will be investigated. The objective of this research is to elucidate the mechanism of lactylation modification in AD, thereby providing new molecular targets for the diagnosis and treatment of AD.

## Materials and methods

### Data acquisition

The GSE153434 and GSE52093 datasets were sourced from the Gene Expression Omnibus (GEO) database for analysis. The GSE153434 dataset, based on the GPL20795 sequencing platform, comprises 10 AD samples and 10 control samples serving as the training set. The GSE52093 dataset, based on the GPL10558 sequencing platform, consists of 7 AD samples and 5 control samples, functioning as the validation set. In addition, 332 lactylation-related genes were extracted from existing literature for this study^25^.

### Differential expression analysis and functional enrichment analysis

The GSE153434 and GSE52093 datasets were normalized using the “Limma” and ‘SVA’ packages in R. *For GSE153434 and GSE52093 datasets*,* quantile normalization was applied using the ‘normalizeBetweenArrays’ function from the ‘Limma’ package to ensure comparability across samples. Additionally*,* log2-transformation was performed on expression values to stabilize variance and approximate a normal distribution.* Differential expression analysis was performed on AD and control samples in the GSE153434 and GSE52093 datasets using the ‘Limma’ package. *To control for false discovery*,* the Benjamini–Hochberg method was applied for multiple testing correction*,* and genes with an adjusted p-value (FDR) < 0.05 and |log2FC| > 1 were considered differentially expressed genes (DEGs). For enrichment analysis*,* Gene Ontology (GO) annotations were obtained from the R package ‘org.Hs.eg.db’ (version 3.17.0)*,* which was also used to define the background gene set. DEGs identified in the training set were mapped to this background for GO enrichment analysis. Kyoto Encyclopedia of Genes and Genomes (KEGG) pathway annotations were retrieved using the KEGG REST API*,* and KEGG enrichment was performed using the ‘clusterProfiler’ package in R*^26–28^. *Terms or pathways with an adjusted p-value (FDR) < 0.05 were considered significantly enriched.*

### Lactylation-associated differentially expressed genes and PPI network

An intersection analysis was conducted between lactylation-associated genes and DEGs in the GSE153434 dataset, to identify lactylation-related differentially expressed genes(LR-DEGs) in AD. *The STRING database was utilized to construct the protein–protein interaction (PPI) network among proteins encoded by the LR-DEGs*,* with a confidence score threshold of 0.7 applied to include only reliable interactions. This network illustrates potential physical interactions*,* regulatory associations*,* and functional relationships among the identified proteins.*

### WGCNA analysis and machine learning to screen key genes

The Weighted Gene Co-expression Network Analysis (WGCNA) was utilized to construct a weighted co-expression network based on gene expression data from the GSE153434 dataset. *To reduce noise and enhance robustness*,* the top 25% of genes with the highest median absolute deviation (MAD) were selected for subsequent analysis. An appropriate soft-thresholding power (β) was determined to ensure scale-free network topology. The resulting adjacency matrix was transformed into a Topological Overlap Matrix (TOM)*,* and gene modules were identified using the dynamic tree cut algorithm. Module–trait correlations were calculated to assess the association between each module and AD. The module showing the strongest correlation (the highest absolute correlation coefficient and lowest p-value) with AD was selected as the key module*,* and the genes within this module were subjected to further analysis.To identify potential diagnostic biomarkers*,* the least absolute shrinkage and selection operator (LASSO) regression was applied using the ‘glmnet’ package in R. A 10-fold cross-validation strategy was used to determine the optimal penalty parameter (lambda) by minimizing the mean cross-validated error. Genes with non-zero coefficients in the optimal model were retained for downstream analysis.*The Random Forest (RF) method is among the most widely used approaches for addressing various predictive challenges. *Hyperparameter tuning*,* including the number of trees and maximum tree depth*,* was performed using grid search combined with 5-fold cross-validation. Model performance was evaluated using several metrics*,* including area under the ROC curve (AUC)*,* accuracy*,* precision*,* recall*,* and F1-score. Feature importance was assessed based on the mean decrease in accuracy*,* and the top-ranking genes were identified as key diagnostic markers for AD.*

## Immune infiltration analysis

CIBERSORT is an algorithm grounded in the principles of linear support vector regression, designed to estimate the abundance of immune cells within a sample. It employs a reference matrix that encompasses gene expression signatures for 22 immune cell subtypes. By contrasting the gene expression profile of a given sample with these established cell types, the algorithm can accurately estimate the proportion of each immune cell type present in the sample.

### Single-cell sequencing analysis

The single-cell transcriptome expression dataset GSE213740, which includes scRNA-seq data from 6 AD patients and 3 normal samples, was sourced from the GEO database. The single-cell transcriptome expression matrix for each sample was generated using the ‘Seurat’ package. *To ensure high-quality data*,* cells with > 15% mitochondrial gene content and genes expressed in fewer than 200 cells were excluded. After this quality control step*,* approximately 20% of low-quality cells were removed. The remaining data were then normalized*,* and batch effects across samples were corrected using the ‘Harmony’ package. Dimensionality reduction was performed using Uniform Manifold Approximation and Projection (UMAP)*,* and cell-type annotation was automatically carried out using the ‘SingleR’ package*,* with results subsequently visualized.*

### Tissue collection from patients

The pathological aortic tissue was collected from patients diagnosed with AD at Renmin Hospital of Wuhan University between 2023 and 2024. The inclusion criteria were as follows: (1) All patients were clinically diagnosed with AD based on imaging methods such as CT and angiography, in conjunction with their clinical symptoms and signs; (2) All patients underwent surgical treatment within two weeks of diagnosis; (3) Patients met the surgical indications and had no contraindications for surgery; (4) Patients were conscious and voluntarily consented to surgical treatment and follow-up. The exclusion criteria included: (1) Patients who did not provide informed consent; (2) Patients with severe coagulopathy; (3) Patients with any type of benign or malignant tumor; (4) Patients with hematological diseases; (5) Patients with a history of drug or alcohol dependence; (6) Patients presenting with delirium, coma, or other cognitive or consciousness impairments upon admission. *(7) Patients with a confirmed history of other major cardiovascular diseases*,* such as coronary artery disease*,* myocardial infarction*,* heart failure*,* arrhythmia*,* or vasculitis*,* to reduce potential confounding effects.* Control group tissue was obtained from healthy aortic tissue of heart transplant donors during the same period. Table S1 presents the demographic and baseline characteristics of patients with aortic disease and the donor group. All trial protocols were conducted following the Declaration of Helsinki and approved by the Human Research Ethics Committees of Renmin Hospital of Wuhan University (WDRY2020-K230) (Wuhan, China). All study subjects or their guardians received verbal and written information about the study and signed a written informed consent form before participation. All aortic specimens were removed from the adventitia and intima and cryopreserved in liquid nitrogen.

### AD model in mice

Twenty 3-week-old male C57BL/6 mice (from Laboratory Animal Center of Guangdong Medical University) were housed in temperature-controlled cages maintained at 20 °C to 25 °C, with unrestricted access to food and water. The mice were randomly assigned to two groups using a digital randomization table: the control group (*N* = 10) and the AD group (*N* = 10). The AD group was administered with a diet containing 0.25% β-aminopropionitrile (BAPN), while the control group received a standard chow diet. Mice were periodically monitored for survival status, and any deceased mice were promptly dissected. Aortic tissue from the dissection site was collected, fixed in 4% paraformaldehyde for 48 h, and subsequently embedded in paraffin for sectioning. Following a modeling period of 4 weeks, all surviving mice were euthanized via spinal cord transection, and tissues were immediately harvested. All trial protocols were approved by the Laboratory Animal Welfare & Ethics Committee of Dongguan Songshan Lake Central Hospital. The experiments involving live vertebrates were carried out in strict accordance with the relevant guidelines and regulations. All methods are reported in accordance with ARRIVE guidelines.

### Western blotting

After washing the aortic tissues with PBS, add lysis buffer prepared with proteinase inhibitors and RIPA strong lysis buffer. Homogenize the tissue on ice for 30 min using a liquid nitrogen grinder. Centrifuge at 4 °C, 12,000 rpm for 20 min using a cold high-speed centrifuge. After centrifugation, collect the supernatant and measure the protein concentration using the BSA kit. Add an appropriate amount of 5× *SDS-PAGE protein* loading buffer to the remaining supernatant, and heat at 95 °C in a metal bath for 10 min. Perform electrophoresis of the protein samples using sodium dodecyl sulfate polyacrylamide gel electrophoresis (SDS-PAGE). *For protein transfer*,* a constant current of 200 mA was applied for 1 h* and block with 5% non-fat dry milk for 2 h. After blocking, wash the membrane 5 times with TBST solution, each for 6 min. Then incubate with primary antibodies: anti-PGK1 (17811-1-AP, Proteintech, China, 1:1000), anti-HMGA1 (29895-1-AP, Proteintech, China, 1:1000), and anti-GAPDH (10494-1-AP, Proteintech, China, 1:5000) at 4 °C for at least 12 h. After the blocking period, wash the membrane 5 times with TBST, each for 6 min. Next, add the corresponding secondary antibody working solution and incubate for 2 h at room temperature. After incubation, wash the membrane 3 times with TBST, each for 6 min. Develop the protein bands using a chemiluminescence imaging system (Bio-Rad, USA) and detect the expression of the proteins.

### Hematoxylin and eosin (HE) staining

*Aortic tissues were fixed*,* dehydrated*,* cleared*,* and embedded in paraffin. Sections were cut at a thickness of 4 μm and mounted on glass slides. Paraffin was removed by immersing the slides in xylene twice for 15 min each. The sections were then rehydrated through a graded ethanol series (100%*,* 95%*,* 80%*,* and 70%) and stained with hematoxylin for 10 min. Differentiation was performed using 1% hydrochloric acid ethanol for 10–60 s*,* followed by rinsing in water and blueing in 0.5% sodium bicarbonate solution. Afterward*,* the sections were counterstained with eosin for 3 min*,* dehydrated in 95% and 100% ethanol*,* cleared in xylene*,* and mounted with coverslips. Histological images were captured using a light microscope (Olympus BX53*,* Tokyo*,* Japan).*

### Immunofluorescence

The tissue sections were hydrated by placing them in xylene, anhydrous ethanol, 95% ethanol, 85% ethanol, 75% ethanol, and 50% ethanol. After washing with PBST solution, the cell membrane was disrupted. The sections were blocked with goat serum, followed by incubation with anti-PGK1 (17811-1-AP, Proteintech, China, 1:50) and anti-HMGA1 (29895-1-AP, Proteintech, China, 1:50) primary antibodies overnight *at 4 °C*. After primary antibody incubation, the sections were washed three times, then incubated with CY3 secondary antibody for 2 h *at room temperature*. Finally, DAPI was used for nuclear staining. After applying an anti-quenching fluorescent mounting medium, the sections were mounted and observed under a fluorescence microscope for imaging.

### Statistics

*Appropriate statistical tests were selected based on the type and distribution of the data. For continuous variables*,* either the t-test or Mann–Whitney U test was used*,* depending on the normality of the data. For categorical variables*,* the chi-square test or Fisher’s exact test was applied. We have conducted a post hoc statistical power analysis based on the observed effect sizes to evaluate the adequacy of the sample size. All statistical analyses were conducted using GraphPad Prism 9 or R version 4.3.3*,* with a significance level set at p < 0.05.*

## Results

### Dataset preprocessing and differential expression analysis

**Fig. 1:**
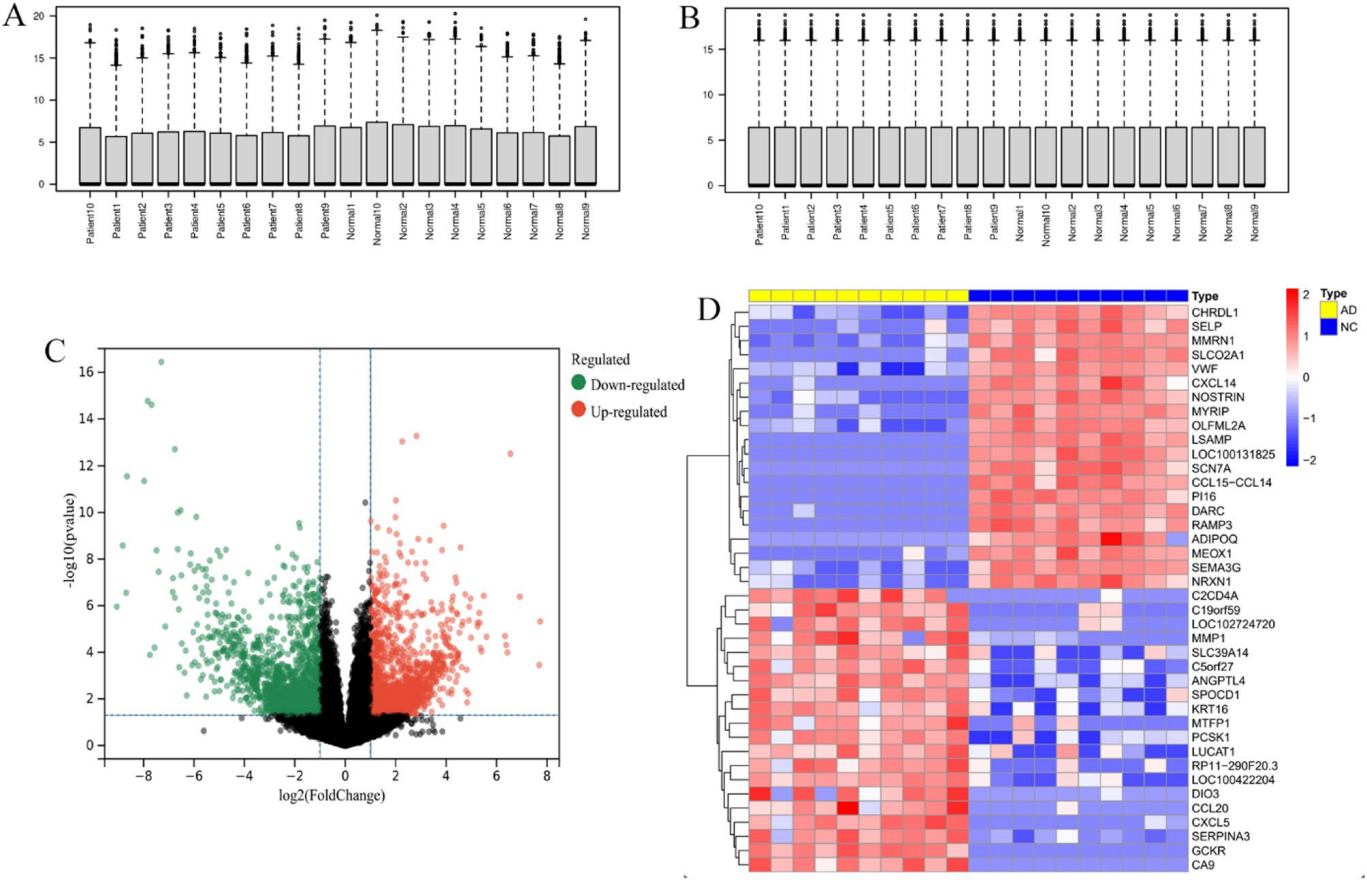
Data Preprocessing and Differential Analysis. (**A-B**) Normalization of expression profiles in the GSE153434 dataset. (**C**) Volcano plot of DEGs in the GSE153434 dataset, with upregulated genes shown in red and downregulated genes shown in green. (**D**) *Heatmap of the top 20 most upregulated and the top 20 most downregulated* DEGs in the GSE153434 dataset.

The expression profile data of the GSE153434 dataset were standardized using the ‘affy’ package. The standardized expression data were then used for subsequent analyses **(**Fig. 1A, B**)**. DEGs between the AD group and the control group were identified using the ‘limma’ package. The criteria for selecting differentially expressed genes were *P* < 0.05 and |logFC| > 1. A total of 3920 differentially expressed genes were identified in the GSE153434 dataset, including 2034 upregulated genes and 1886 downregulated genes, which were visualized using volcano plots and heatmaps **(**Fig. 1C, D**)**.

### Functional enrichment analysis of DEGs and screening of lactylation-related differentially expressed genes

**Fig. 2:**
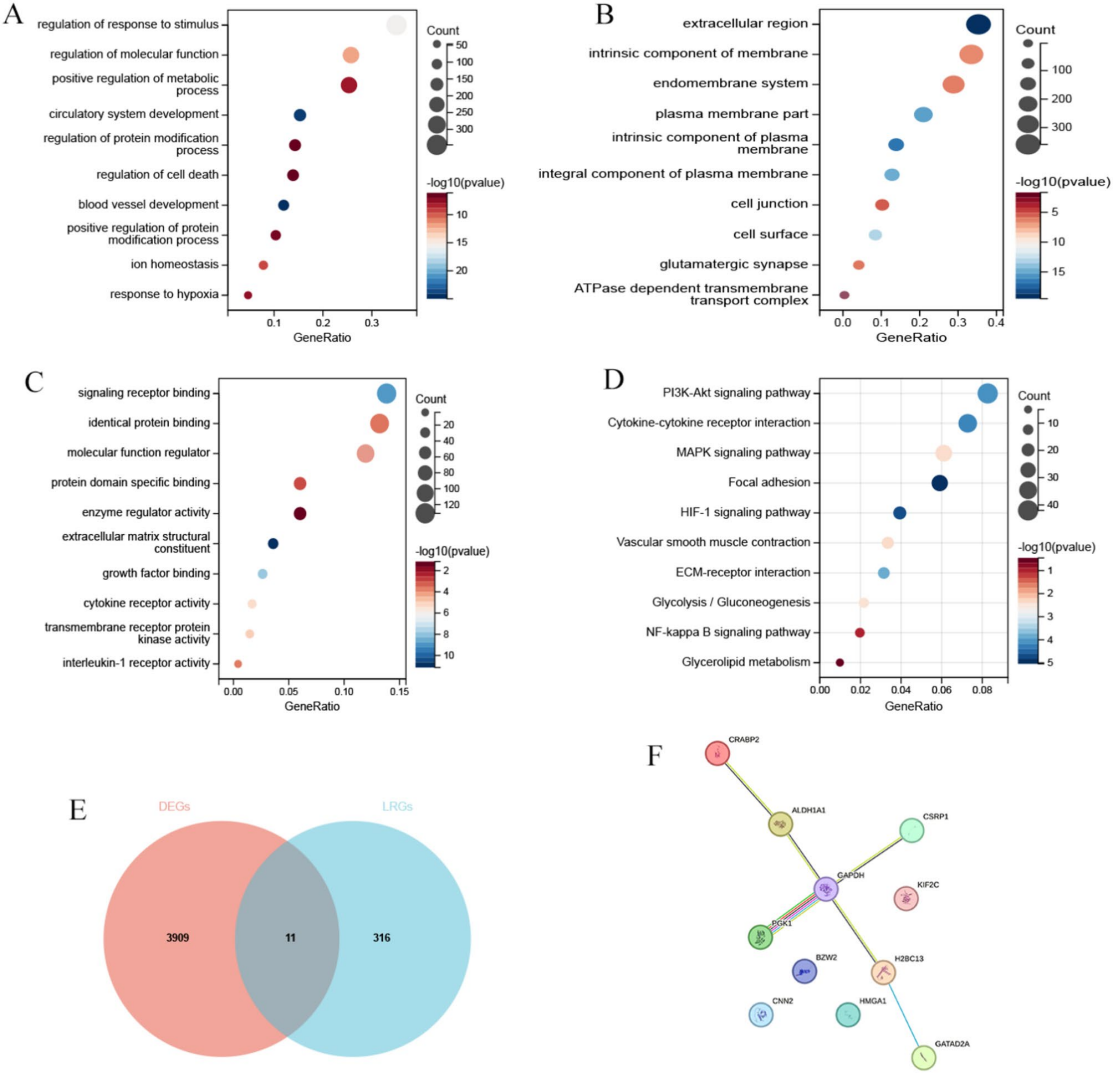
Functional Enrichment and PPI Analysis. (**A**) Biological Process (BP) categories in the GO enrichment analysis of DEGs. (**B**) Cellular Component (CC) categories. (**C**) Molecular Function (MF) categories. (**D**) KEGG pathway enrichment analysis of DEGs. (**E**) Venn diagram showing the overlap between DEGs from the GSE153434 dataset and lactylation-related genes. (**F**) PPI network of lactylation-related DEGs.

A total of 3920 differentially expressed genes were subjected to GO and KEGG enrichment analysis. The GO enrichment analysis indicated that, in terms of biological processes, these DEGs were primarily associated with stress response regulation, hypoxia response, metabolic processes, and cell death **(**Fig. 2A**)**. In terms of cellular components, these genes were widely distributed in the extracellular matrix, cell membrane, and cytoplasm **(**Fig. 2B**)**. Regarding molecular functions, these genes were primarily enriched in signaling pathway receptors, enzyme activity, and cytokine receptors **(**Fig. 2C**)**. The KEGG enrichment analysis revealed that these genes were related to the PI3K-AKT pathway, HIF-1 pathway, NF-κB pathway, glycolysis, and glucose-lipid metabolism, among others **(**Fig. 2D**)**. The intersection of these differentially expressed genes with lactylation-related genes yielded 11 lactylation-related genes that were differentially expressed in aortic dissection. These genes are ALDH1A1, HMGA1, PGK1, GAPDH, CSRP1, CNN2, BZW2, CRABP2, HIST1H2BL, GATAD2A, and KIF2C **(**Fig. 2E**)**. PPI network analysis of these differentially expressed lactylation-related genes suggested significant interactions among these genes **(**Fig. 2F**)**.

### Construction of WGCNA

**Fig. 3:**
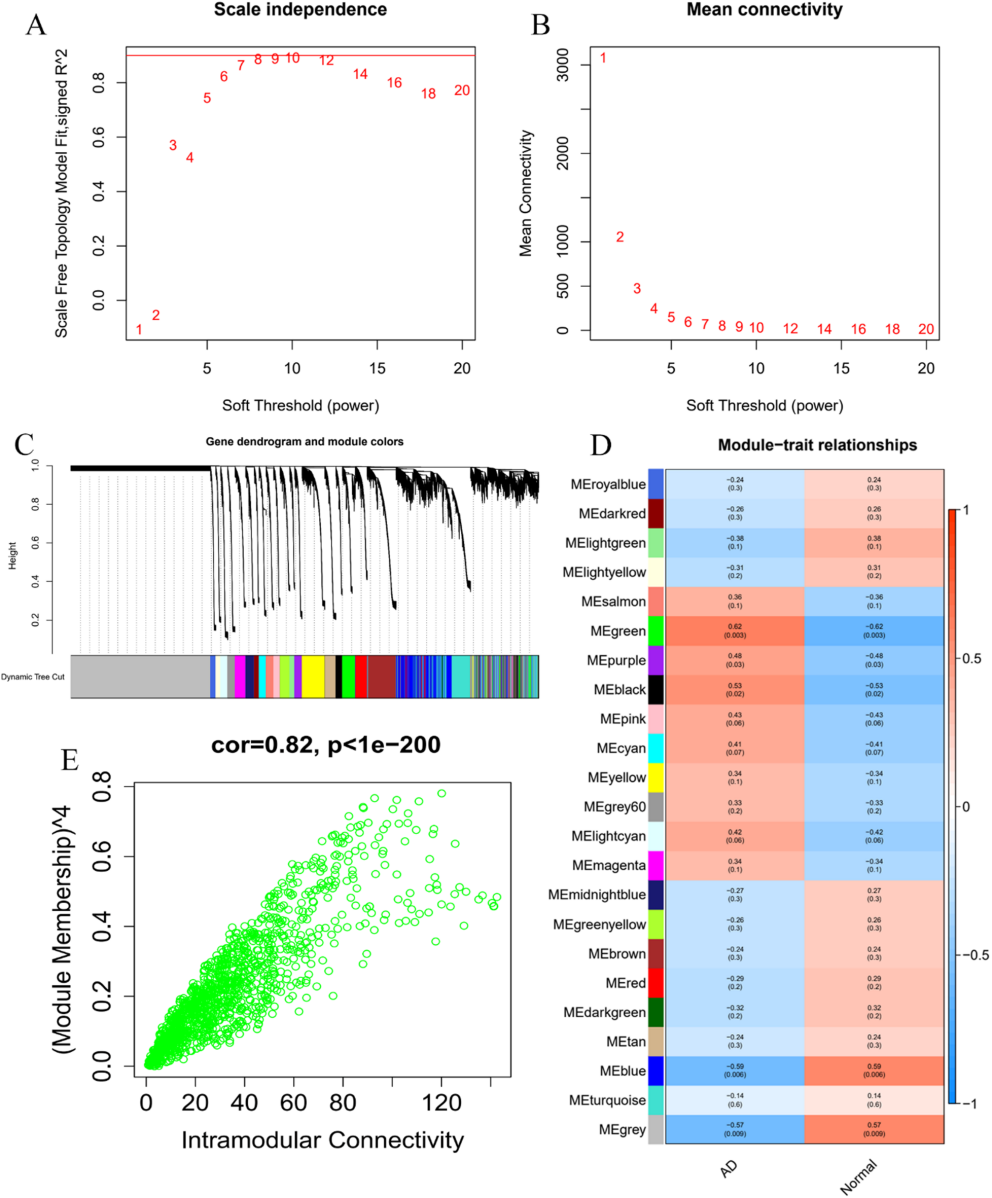
WGCNA Analysis and Key Module Selection.(**A-B**) Determination of the soft threshold for WGCNA analysis.(**C**) Identification of co-expression network modules.(**D**) Heatmap showing the correlation between WGCNA modules and AD.(**E**) Scatter plot of gene-module correlations with AD in WGCNA.

Clustering was conducted on 10 normal samples and 10 AD samples from the GSE153434 dataset, with samples exhibiting significant anomalies excluded based on a predetermined threshold. The soft threshold was set to 8 when R^2^ value exceeded 0.9 and the average connectivity was relatively high **(**Figs. 3A, B**)**. After merging strongly correlated modules with a clustering height limit of 0.25, a total of 23 modules were identified for further investigation. The initiated and merged modules are displayed in the clustering tree **(**Fig. 3C**)**. To explore the relationship between the module eigengene (ME) values and clinical features, a correlation analysis was conducted, revealing a significant association between the modules and clinical symptoms **(**Fig. 3D**)**. The results indicated that in the scatter plot of AD MM and GS, the green module showed a strong correlation with AD. A total of 1277 genes from the green module were extracted for further analysis **(**Fig. 3E**)**.

### Multiple machine learning algorithms to identify optimal feature genes

**Fig. 4:**
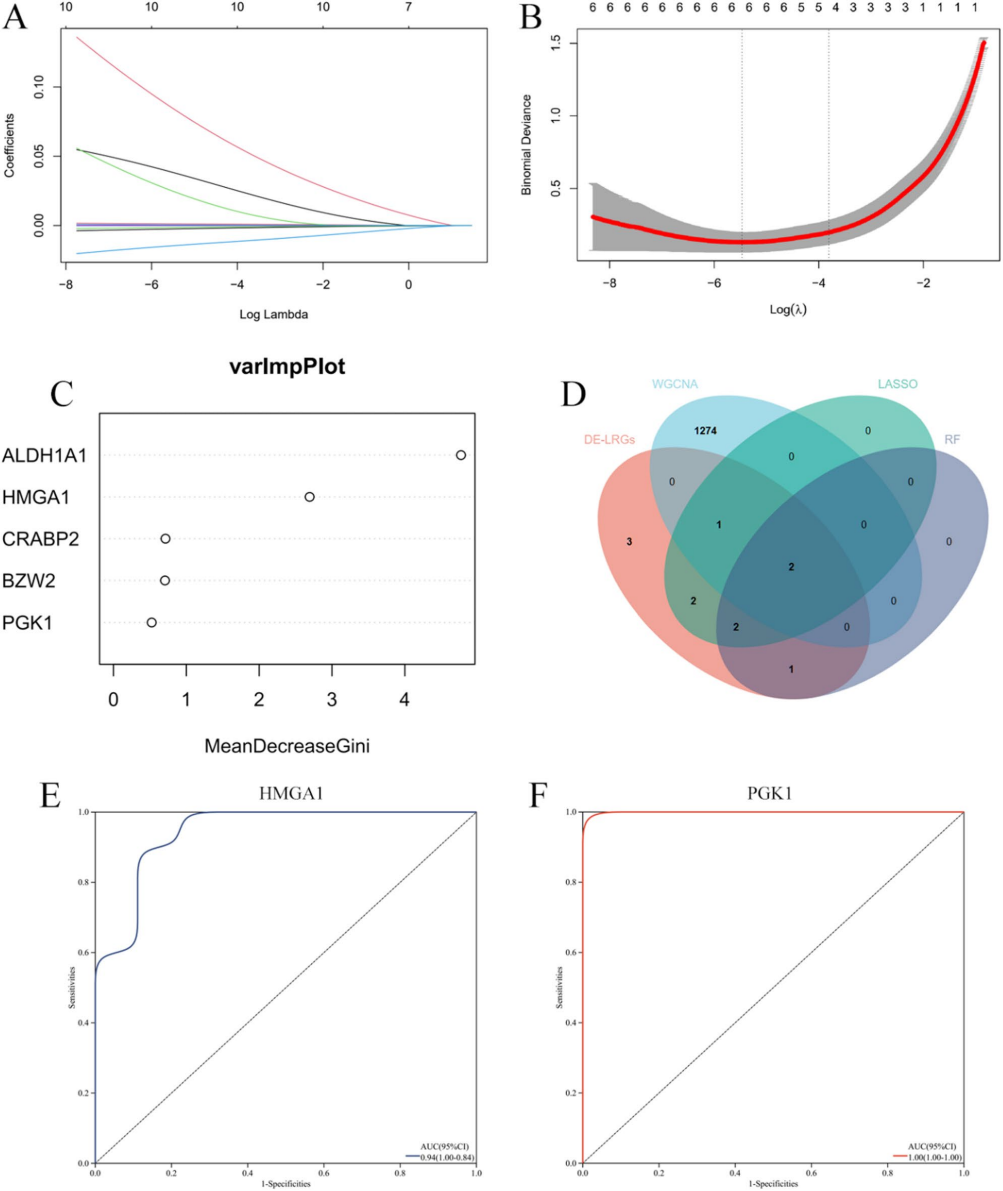
Machine Learning and Optimal Feature Genes Screening.(A-B): LASSO regression identifies biomarkers for AD. *(***A***): The trajectory of gene coefficients as the regularization parameter (log λ) increases*,* where less important gene coefficients shrink toward zero. (***B***): The 10-fold cross-validation curve*,* with the optimal λ value (λ_min) selected based on the minimum binomial deviance to achieve the best model performance with minimal overfitting.* (**C**): RF identifies biomarkers for AD. (**D**): Venn diagram of biomarkers for AD identified by both machine learning and WGCNA. (**E**): ROC analysis showing the diagnostic efficiency of HMGA1 for AD. (F): ROC analysis showing the diagnostic efficiency of PGK1 for AD.

To identify the optimal feature genes, two different machine learning algorithms were applied. Specifically, we used the LASSO analysis algorithm to select 7 feature genes as diagnostic biomarkers for AD from the 11 candidate genes **(**Figs. 4A, B**)**. Concurrently, the SVM-REF algorithm was employed, and after performing 5-fold cross-validation on the 11 candidate feature genes, 5 feature genes were selected **(**Fig. 4C**)**. By intersecting the feature genes identified from these two machine learning algorithms with the genes from the green module in the WGCNA analysis, we identified HMGA1 and PGK1 as the two optimal feature genes **(**Fig. 4D**)**. Furthermore, the diagnostic and predictive value of the optimal feature genes was quantitatively assessed using ROC curves. The results indicated that the AUC value for the ROC curve of the HMGA1 gene was 0.94 **(**Fig. 4E**)**, and the AUC value for the ROC curve of the PGK1 gene was 1 **(**Fig. 4F**)**. These findings suggest that these optimal feature genes are capable of estimating disease progression and possess high diagnostic value for AD.

### External dataset validation of optimal feature genes

**Fig. 5:**
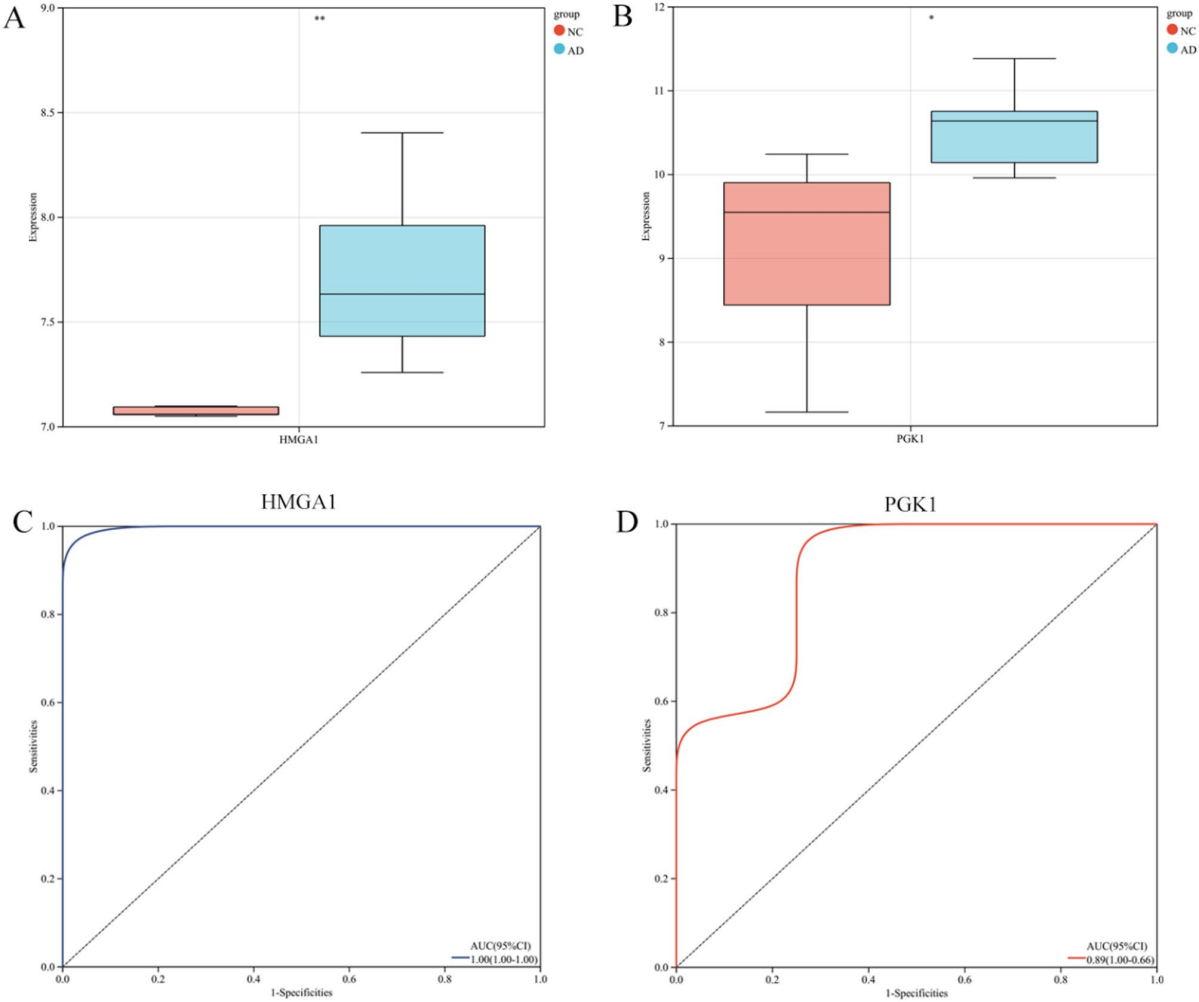
Cross-validation of the optimal feature genes in an external dataset. (**A-B**): Expression levels of HMGA1 and PGK1 in the GSE52093 dataset. (**C-D**): Diagnostic efficiency of HMGA1 and PGK1 in the GSE52093 dataset.

Additionally, we further validated the expression levels of the optimal feature genes in the external validation dataset GSE52093. Compared to control samples, the expression of the two optimal feature genes was significantly upregulated in AD samples **(**Fig. 5A, B**)**. Furthermore, HMGA1 and PGK1 also exhibited high AUC values in the external validation dataset. The AUC value of the HMGA1 gene ROC curve was 1.0 **(**Fig. 5C**)**, while the AUC value of the PGK1 gene ROC curve was 0.89 **(**Fig. 5D**)**. These external validation results strongly support the involvement of these optimal feature genes in AD and highlight their significant diagnostic value for the disease.

### Immune infiltration and the correlation between feature genes and immune infiltration

**Fig. 6:**
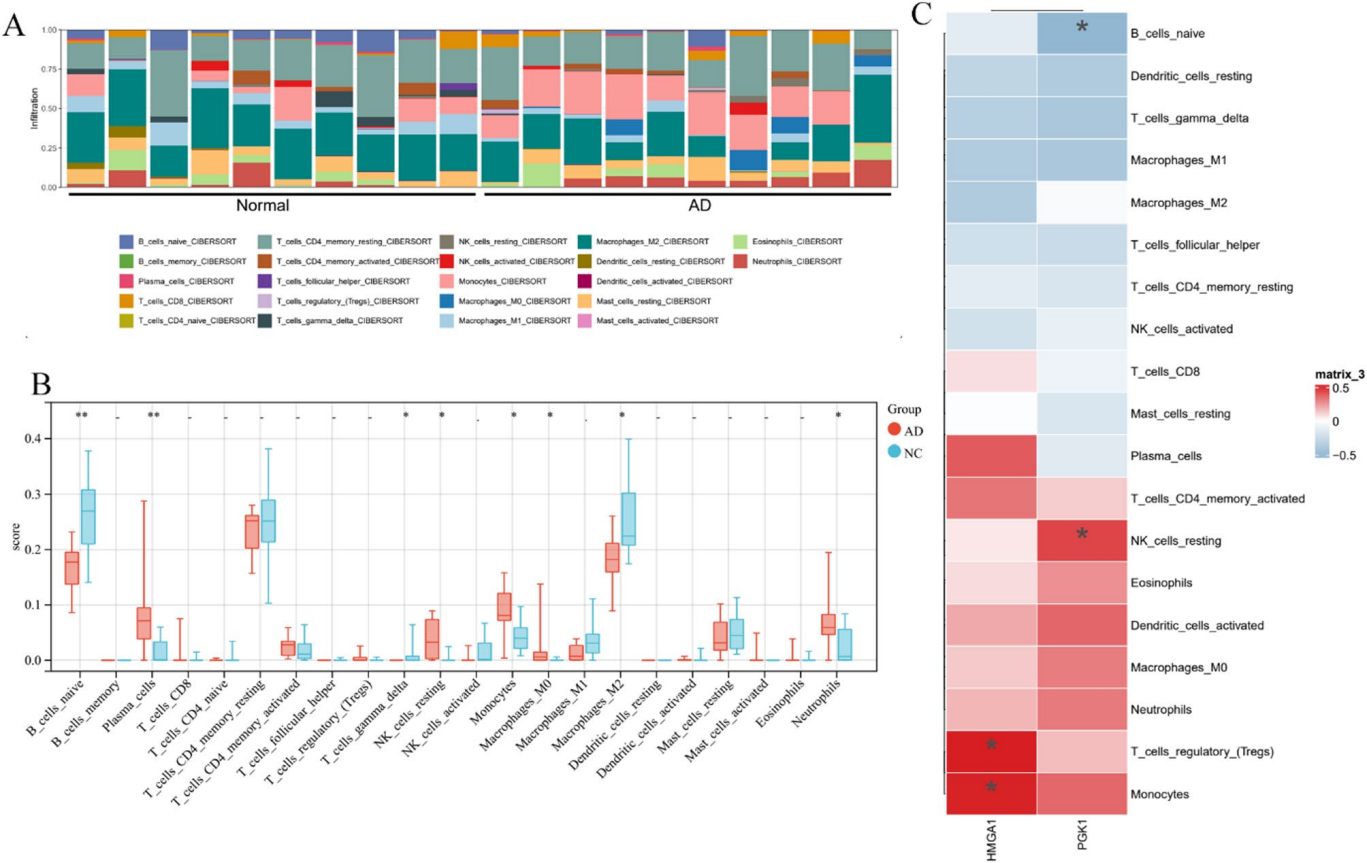
Immune infiltration and correlation analysis. (**A**) Stacked bar plot comparing 22 immune cell subsets between AD and NC samples. (**B**) Bar plot showing the differences in the proportions of 22 immune cell types between AD and NC samples. (**C**) Correlation analysis plots of HMGA1, PGK1, and immune infiltration (positive correlation coefficients indicate positive correlations, while negative coefficients indicate negative correlations).

The CIBERSORT algorithm was employed to further evaluate the differences in immune cell infiltration between AD and control samples. The infiltration abundance of 22 types of immune cells in each sample is depicted in the bar chart **(**Fig. 6A**)**. Compared to control samples, the proportions of plasma cells, resting NK cells, monocytes, M0 macrophages, and neutrophils were significantly increased in AD samples, while the proportions of naïve B cells and M2 macrophages were significantly decreased **(**Fig. 6B**)**. Furthermore, correlation analysis between immune cell types and the genes HMGA1 and PGK1 indicated that HMGA1 was highly positively correlated with monocytes and Tregs, while PGK1 exhibited a strong positive correlation with resting NK cells and a negative correlation with naïve B cells **(**Fig. 6C**)**. These results suggest a potential link between HMGA1 and PGK1 and immune cell infiltration.

### Single-cell RNA sequencing analysis

**Fig. 7:**
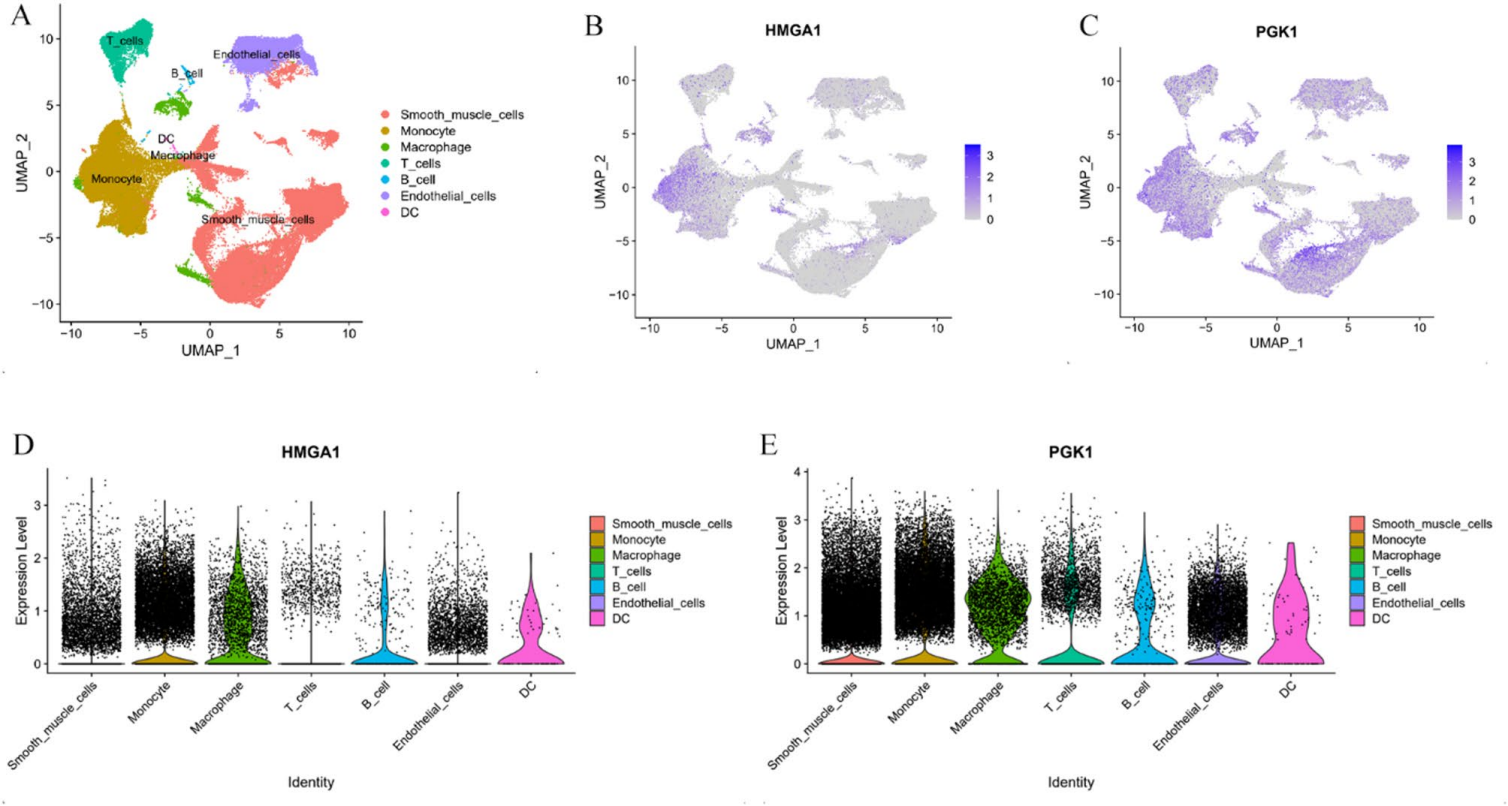
Single-cell sequencing analysis. (**A**): UMAP plot showing cell clustering. (**B**): UMAP plot depicting HMGA1 expression in each cluster. (**C**): UMAP plot depicting PGK1 expression in each cluster. (**D**): Violin plot showing HMGA1 expression across different cell groups. (**E**): Violin plot showing PGK1 expression across different cell groups.

Hierarchical clustering was used to divide the cells into seven clusters, which were identified based on marker genes as Smooth muscle cells, Monocytes, Macrophages, Endothelial cells, B cells, T cells, and Tissue stem cells **(**Fig. 7A**)**. The expression patterns of HMGA1 and PGK1 across different cell populations in AD tissues were analyzed. The results indicated that the HMGA1 gene is primarily expressed in Monocytes **(**Fig. 7B, D**)**. The PGK1 gene is primarily expressed in Smooth muscle cells, Monocytes, and Endothelial cells **(**Fig. 7C, E**)**.

### Aortic tissue validation in patients

**Fig. 8:**
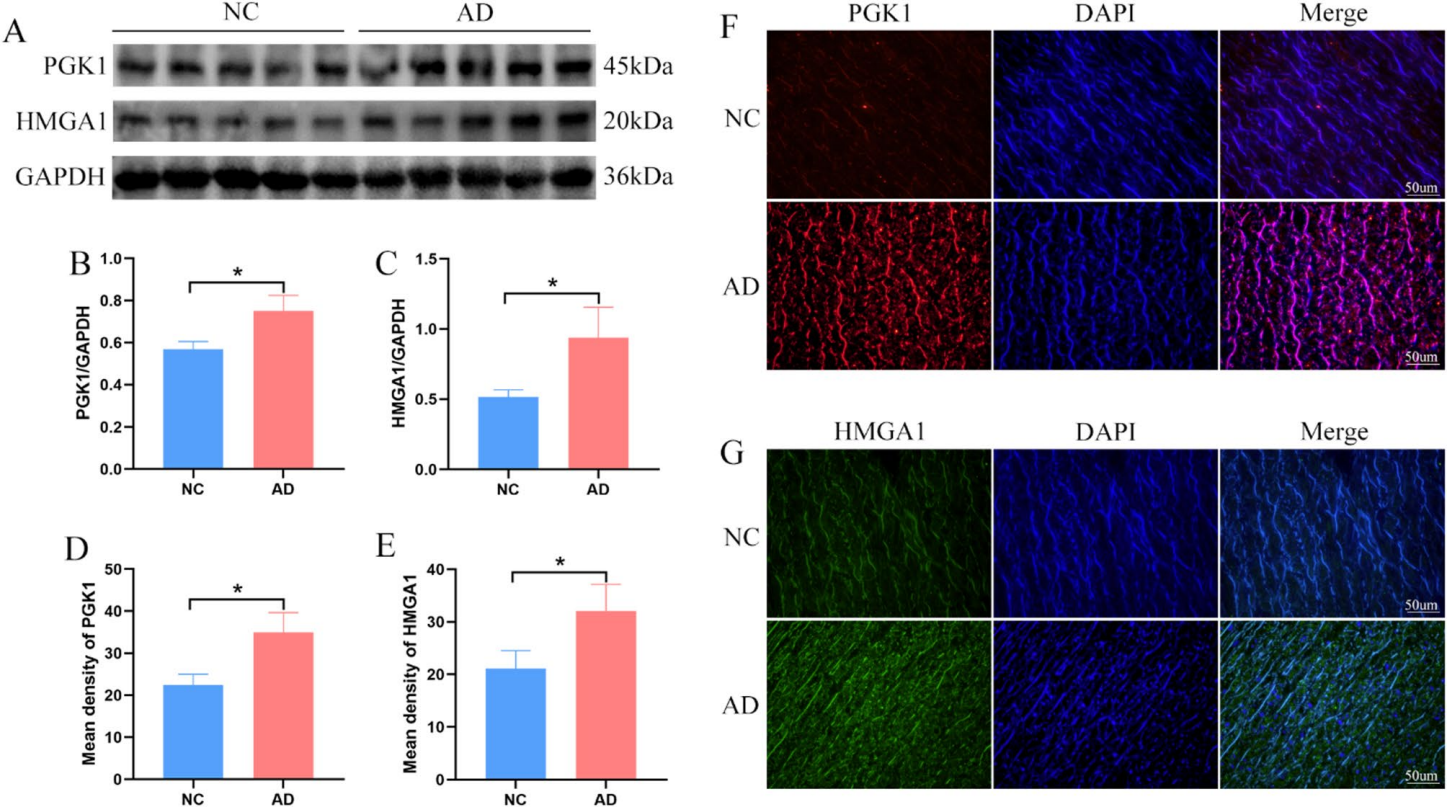
Validation of Optimal Feature Gene Expression in Patient Aortic Tissues. (**A-C**): Western blotting analysis of HMGA1 and PGK1 expression in aortic tissues from AD and NC groups, along with corresponding results. (**D-G**): Immunofluorescence analysis of HMGA1 and PGK1 expression in aortic tissues from AD and NC groups, along with corresponding results.

Western blotting was performed to detect the expression levels of PGK1 and HMGA1 in aortic tissue from patients with AD and a control group. The results indicated that compared to the control group, the expression levels of PGK1 and HMGA1 were significantly upregulated in the aortic tissue of patients with AD **(**Fig. 8A-C**)**. Furthermore, immunofluorescence experiments were conducted to further investigate the in situ expression of PGK1 and HMGA1 in aortic tissue, and the results similarly demonstrated that the expression levels of PGK1 and HMGA1 were upregulated in the aortic tissue of patients with AD **(**Fig. 8D-G**)**.

### Animal model validation

**Fig. 9:**
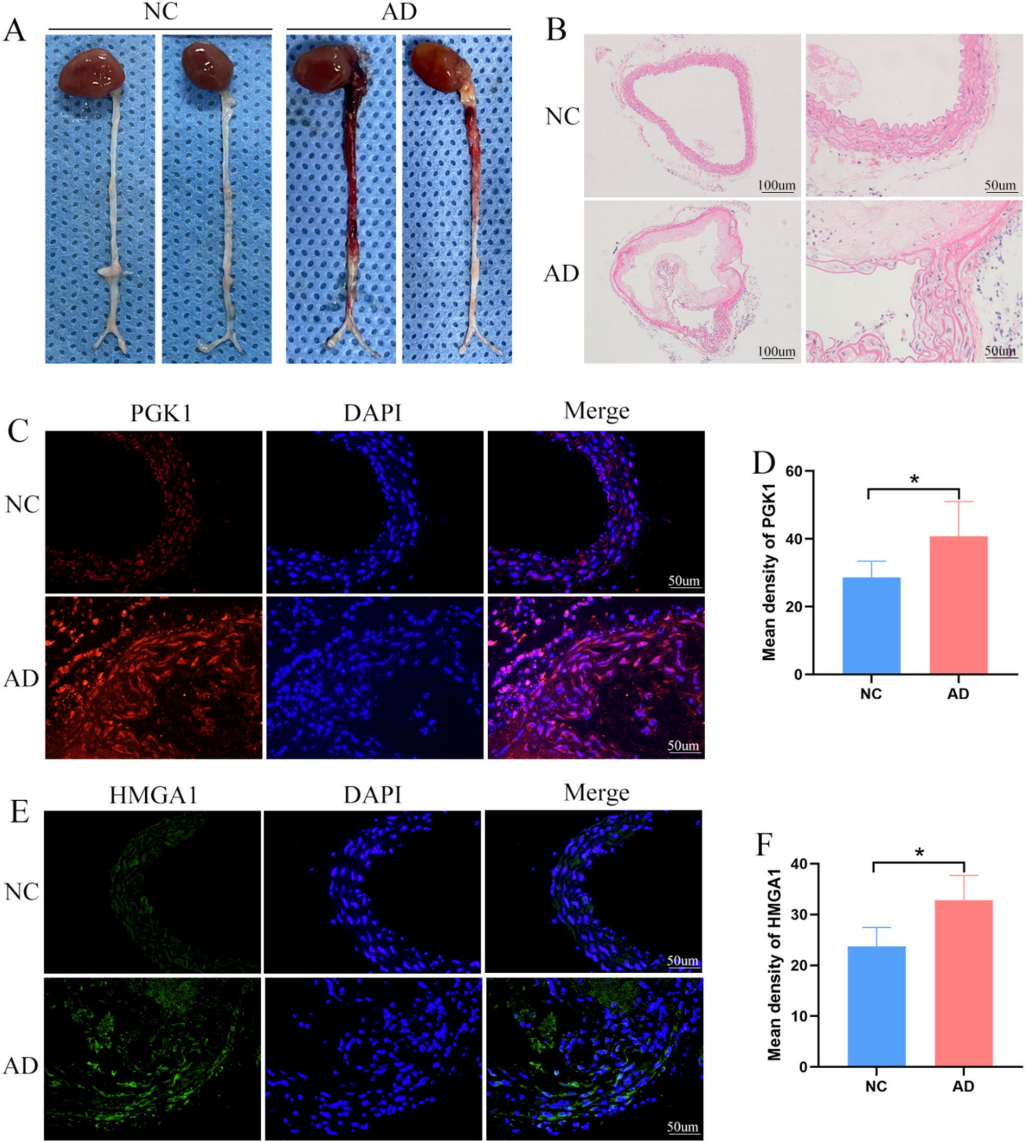
Validation of the expression of optimal feature genes in animal experiments. (**A**) Gross appearance of the aorta in the control group and AD group mice. (**B**) Histopathological observations of the aorta in the control group and AD group mice. (**C-D**) Expression levels and statistical analysis of PGK1 in the aorta of the control group and AD group mice. (**E-F**) Expression levels and statistical analysis of HMGA1 in the aorta of the control group and AD group mice.

Animal model was established using BAPN to further investigate the expression of PGK1 and HMGA1 in AD. The gross image of the aorta showed the formation of aortic dissection **(**Fig. 9A**)**. HE staining was performed to examine the pathological features of the AD in the mice **(**Fig. 9B**)**. Immunofluorescence staining was used to detect the expression of PGK1 and HMGA1 in both the AD mice and control groups. The results indicated that PGK1 and HMGA1 were significantly overexpressed in the AD mice model **(**Figs. 9C-F**)**.

## Discussion

Aortic dissection is a severe cardiovascular condition characterized by a tear in the inner layer of the aorta, allowing blood to enter the space between the intima and media, forming a dissection. The occurrence of AD is frequently associated with local hypoxia and metabolic abnormalities^29,30^. The role of hypoxia in this disease has garnered increasing attention, with lactylation—a post-translational modification of proteins—being closely associated with hypoxia and metabolic changes^31^. Lactylation is an emerging protein modification in which lactate molecules, typically derived from glycolysis, bind to target proteins, akin to acetylation or methylation^32^. This modification is particularly significant under hypoxic conditions because, in hypoxia or high-glycolysis environments, cells accumulate lactate, which not only serves as a metabolic byproduct but may also function as a signaling molecule that influences protein activity^31^. Hypoxia results in elevated intracellular lactate levels, which, through the process of lactylation, influences numerous critical cellular functions. Specifically, lactylation plays a regulatory role in proteins associated with cell proliferation, metabolism, apoptosis, and migration^33,34^. In the context of aortic dissection, the enhancement of glycolysis and accumulation of lactate induced by hypoxia may regulate intracellular signaling pathways through lactylation, thereby influencing the function of vascular smooth muscle cells^35^. This regulatory effect may lead to vascular wall remodeling, increased expression of matrix metalloproteinases, and even compromise the structural integrity of the vessel wall, further exacerbating the progression of AD^35^.

This study investigates the expression of lactylation-related genes in AD and aims to identify optimal feature genes, exploring their potential roles and diagnostic value in the disease. Transcriptomic expression data were downloaded from the GEO database, and differential analysis was performed to identify DEGs. GO and KEGG functional enrichment analyses revealed that these DEGs are enriched in glycolysis and lactylation-related pathways. Previous studies have similarly demonstrated differential expression of certain glycolysis and lactylation-related genes in AD^35,36^. Further intersection of these DEGs with lactylation-related genes identified the lactylation-related genes that are differentially expressed in AD. *As shown in the PPI network*,* GAPDH appears as a central hub interacting with several candidate biomarkers*,* including PGK1*,* ALDH1A1*,* CRABP2*,* and HMGA1. This suggests that GAPDH may play a central regulatory role in the metabolic or stress-related pathways associated with AD. Notably*,* PGK1 and HMGA1*,* which were identified as key diagnostic markers by machine learning*,* also show direct or indirect interactions in the network*,* implying their involvement in interconnected biological processes such as glycolysis*,* cell proliferation*,* and inflammatory responses. These functional associations may contribute to the vascular remodeling and pathological changes observed in AD.* Next, WGCNA was conducted to screen for gene modules most correlated with AD, and genes from these modules were extracted for further analysis. LASSO and RF machine learning methods were then applied to further select lactylation modification feature genes associated with AD. Finally, by intersecting the results from machine learning and WGCNA, two genes, PGK1 and HMGA1, were identified as being most related to lactylation in AD.

PGK1 is a crucial enzyme in cellular energy metabolism, primarily catalyzing the conversion of 3-phosphoglycerate to 1,3-bisphosphoglycerate, thereby participating in the glycolytic pathway^37^. Recent studies have demonstrated that PGK1 not only plays an essential role in energy metabolism but also regulates various cellular functions, including the modulation of protein lactylation^38^. During the development of AD, the lactylation regulation of PGK1 may play a crucial role in the structural and functional changes of the aorta by affecting the metabolic state and function of smooth muscle cells. PGK1 may influence the metabolic pathways of aortic smooth muscle cells, thereby regulating processes such as cell proliferation, migration, and apoptosis, which in turn affect the stability and elasticity of the aortic wall, thereby increasing the risk of AD^39^. HMGA1 is a non-histone transcription factor that primarily participates in biological processes such as cell proliferation, differentiation, migration, and stress response through binding to DNA and regulating gene expression^40^. Studies have found that HMGA1 not only plays a crucial role in cellular functions but may also be significantly involved in the regulation of cell metabolism and gene expression through post-translational modifications^41^. The results of this study indicate a significant correlation between HMGA1 and monocytes, suggesting that HMGA1 may influence the metabolic activities and functional changes of immune cells through its lactylation modification, thus participating in the structural remodeling and stability regulation of the aortic wall^42^. HMGA1 lactylation may affect gene expression related to cellular stress, inflammatory responses, and metabolic regulation, thereby influencing immune functions^43^. *In addition to PGK1 and HMGA1*,* several other lactylation-related genes identified in our analysis*,* such as GAPDH and ALDH1A1*,* may also contribute to the pathophysiological mechanisms of aortic dissection. GAPDH*,* classically known for its role in glycolysis*,* has been increasingly recognized as a multifunctional protein involved in nuclear signaling*,* oxidative stress response*,* and inflammation*^44,45^. *Its lactylation may modulate these non-metabolic functions*,* potentially influencing vascular inflammation and immune cell recruitment in AD. Similarly*,* ALDH1A1 plays a critical role in retinoic acid metabolism and cellular detoxification*^46^. *The lactylation of ALDH1A1 could affect its enzymatic activity and downstream signaling*,* thereby contributing to extracellular matrix degradation and vascular smooth muscle cell dysfunction. These findings suggest that GAPDH and ALDH1A1*,* through lactylation-mediated mechanisms*,* may represent additional regulatory nodes in the progression of aortic dissection and warrant further experimental investigation.*

By examining the expression of PGK1 and HMGA1 in the aortic tissues of patients with AD and a control group, our results indicated that the expression levels of PGK1 and HMGA1 were significantly elevated in AD patients compared to the normal control group. This finding suggests that PGK1 and HMGA1 may contribute significantly to the pathogenesis of AD. Subsequently, a mouse model of AD induced by BAPN was established, and the expression levels of PGK1 and HMGA1 in the mouse aorta were examined, yielding results consistent with those observed in human tissue. Specifically, the expression levels of PGK1 and HMGA1 in the mouse AD model were significantly elevated compared to the control group. In conclusion, both bioinformatics analysis and experimental validation support the notion that PGK1 and HMGA1 play crucial roles in AD, with their mechanisms potentially linked to protein lactylation.

### Limitations

*This study has several limitations. The relatively small sample sizes used in both the public datasets and our experimental validation may limit the statistical power and generalizability of the findings. We acknowledge this limitation and suggest that future studies should include larger*,* independent cohorts and consider statistical power analysis to improve the robustness and interpretability of the results. Although PGK1 and HMGA1 were identified as potential key regulators associated with lactylation in aortic dissection based on bioinformatic analysis*,* their mechanistic roles were not directly validated through functional experiments. Further in vitro and in vivo studies are needed to confirm their biological functions*,* particularly in relation to lactylation-specific modifications. In addition*,* although CIBERSORT was employed to estimate immune cell infiltration from bulk RNA-seq data*,* it has inherent limitations*,* especially in distinguishing closely related immune cell subtypes and capturing disease-specific immune heterogeneity. To enhance the accuracy and resolution of immune deconvolution*,* future studies could consider integrating additional tools such as xCell or EPIC. These complementary approaches may provide a more comprehensive and nuanced understanding of the immune microenvironment in aortic dissection. Furthermore*,* although we identified key lactylation-related genes and enriched pathways associated with AD*,* the study did not explore potential upstream regulatory mechanisms*,* such as transcription factors or epigenetic modulators*,* that may drive these gene expression changes. This represents an important direction for future work*,* and integrative multi-omics approaches will be required to uncover the regulatory landscape underlying lactylation dynamics in AD.*

## Conclusion

This study reveals a correlation between lactate metabolism and AD through a combination of bioinformatics and experimental validation. The PGK1 and HMGA1 genes were identified as diagnostic biomarkers for AD. Furthermore, enrichment analysis, immune infiltration, and single-cell analysis enhanced our understanding of the role of lactylation modification in the pathogenesis of AD. Finally, the validation of bioinformatics results using human samples and animal experiments further corroborated these findings.

## Electronic supplementary material

Below is the link to the electronic supplementary material.

Supplementary Material 1

Supplementary Material 2

## Data Availability

The primary information substantiating the findings of this investigation will be provided by the corresponding author without unnecessary delay or restriction.

## Abbreviations

AD: Aortic dissection
CTA: Computed Tomography Angiography
PTMs: post-translational modifications
GEO: Gene Expression Omnibus
DEGs: differentially expressed genes
GO: Gene Ontology
KEGG: Kyoto Encyclopedia of Genes and Genomes
PPI: Protein-Protein Interaction
WGCNA: Weighted Gene Co-expression Network Analysis
LASSO: Least Absolute Shrinkage and Selection Operator
RF: Random Forest
BAPN: β-aminopropionitrile
