# Supplementary figures and images for: Lactylation associated biomarkers and immune infiltration in aortic dissection
Source: Sci Rep. 2025 Jul 1;15:21536. doi: 10.1038/s41598-025-08613-y (PMC12219385; doi:10.1038/s41598-025-08613-y)

PGK1

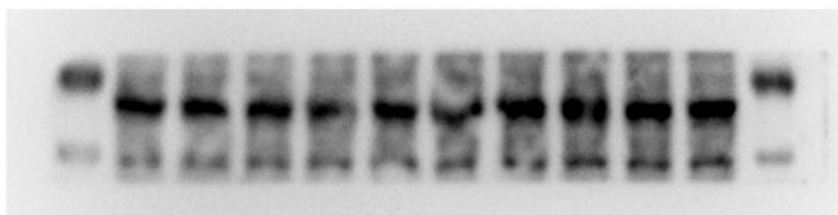

45kDa

GAPDH

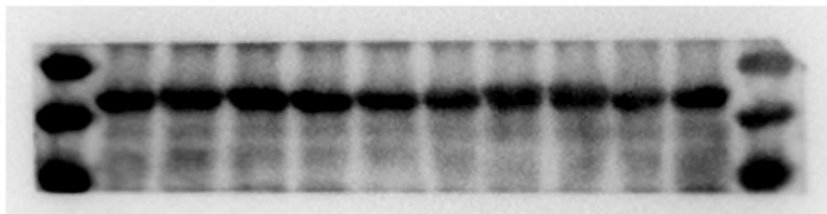

36kDa

GAPDH

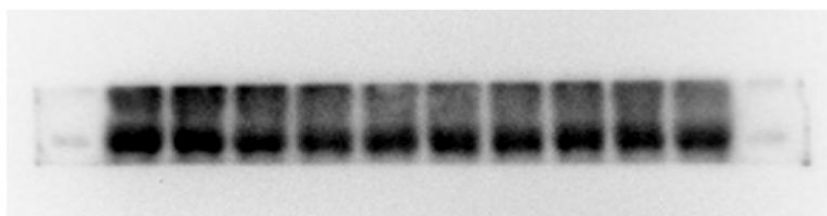

36kDa

HMGA1

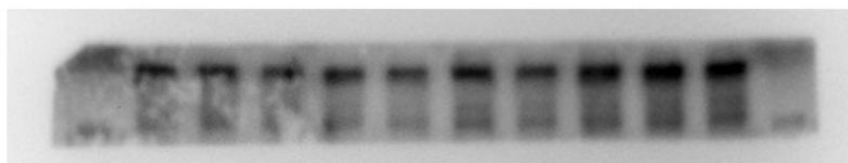

20kDa

Supplement: Supplementary file 1 — Supplementary Material 1 [file 41598_2025_8613_MOESM1_ESM.pdf]
