# Supplementary material for: Lactylation associated biomarkers and immune infiltration in aortic dissection
Source: Sci Rep. 2025 Jul 1;15:21536. doi: 10.1038/s41598-025-08613-y (PMC12219385; doi:10.1038/s41598-025-08613-y)
Supplement: Supplementary file 2 — Supplementary Material 2 [file 41598_2025_8613_MOESM2_ESM.docx]

Table S1: Baseline characteristics.

| Parameters | AD Group (n = 12) | Control Group (n = 10) | P value |
| --- | --- | --- | --- |
| Age (years) | 49.35±6.71 | 46.27±5.19 | ns |
| Male/female (n) | 9(75.00) | 7(70.00) | ns |

Values are expressed as mean ± standard deviation or n (%).

Abbreviations: AD, aortic dissection
